# Supplementary figures and images for: Kinetic regulation of kinesin’s two motor domains coordinates its stepping along microtubules
Source: eLife. 2025 Apr 17;14:RP106228. doi: 10.7554/eLife.106228 (PMC12005725; doi:10.7554/eLife.106228)

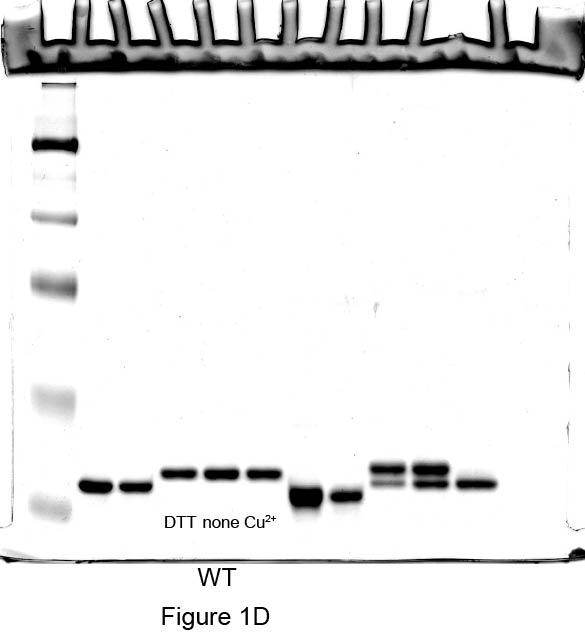

Supplement: Figure 1—source data 1. [file elife-106228-fig1-data1.zip › Fig1D-source1-WT.jpg]

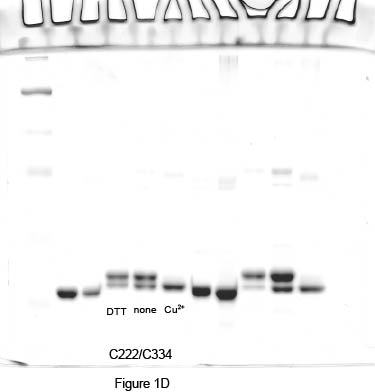

Supplement: Figure 1—source data 1. [file elife-106228-fig1-data1.zip › Fig1D-source1-rear.jpg]

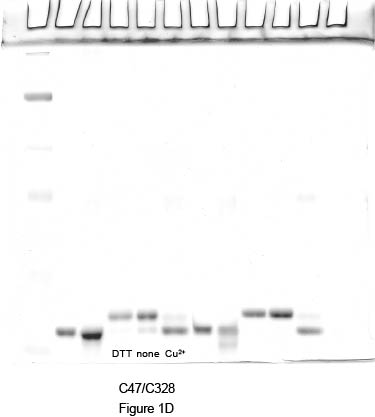

Supplement: Figure 1—source data 1. [file elife-106228-fig1-data1.zip › Fig1D-source1-front.jpg]

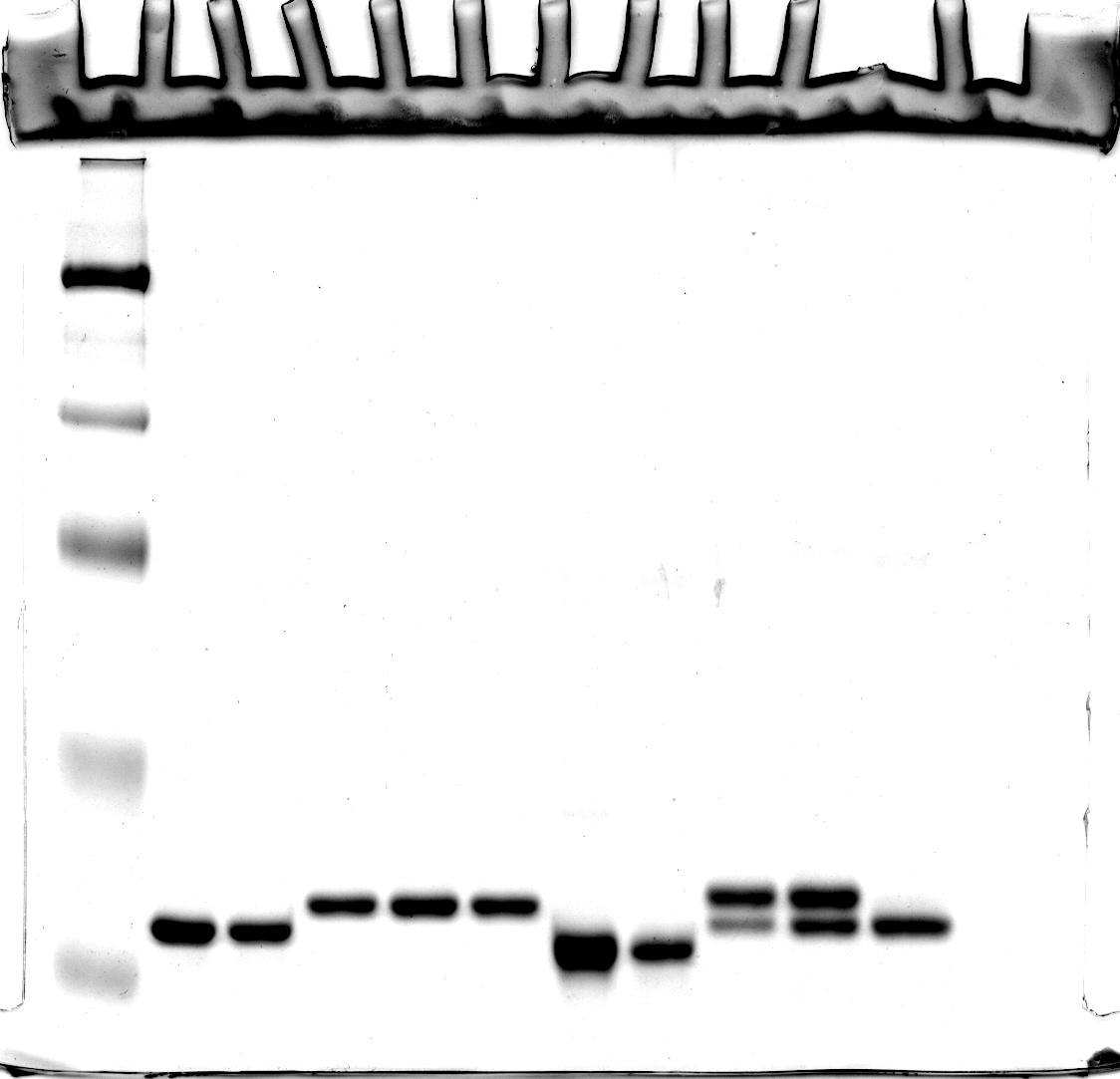

Supplement: Figure 1—source data 2. [file elife-106228-fig1-data2.zip › Fig1D-source2-WT.jpg]

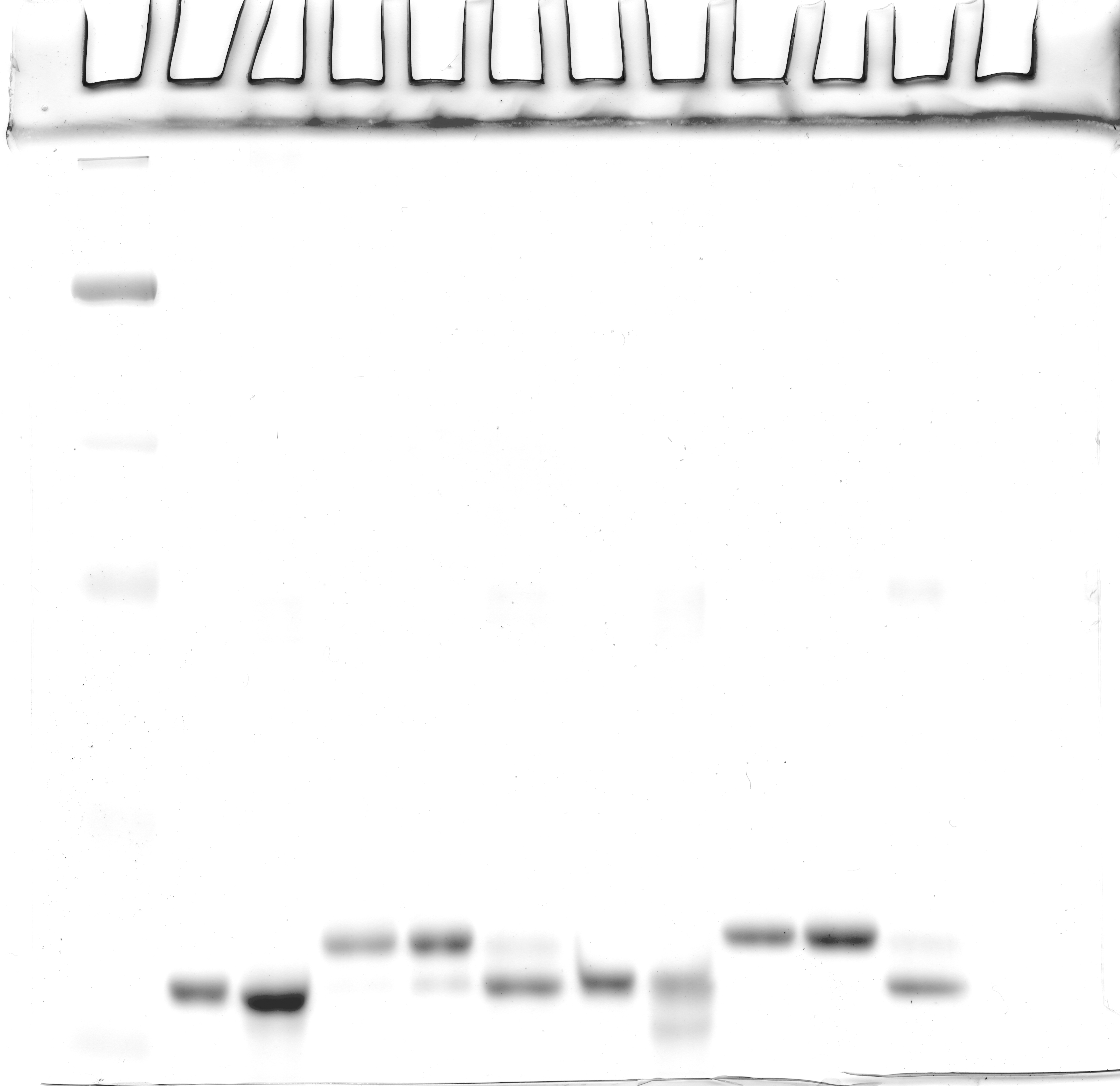

Supplement: Figure 1—source data 2. [file elife-106228-fig1-data2.zip › Fig1D-source2-front.jpg]

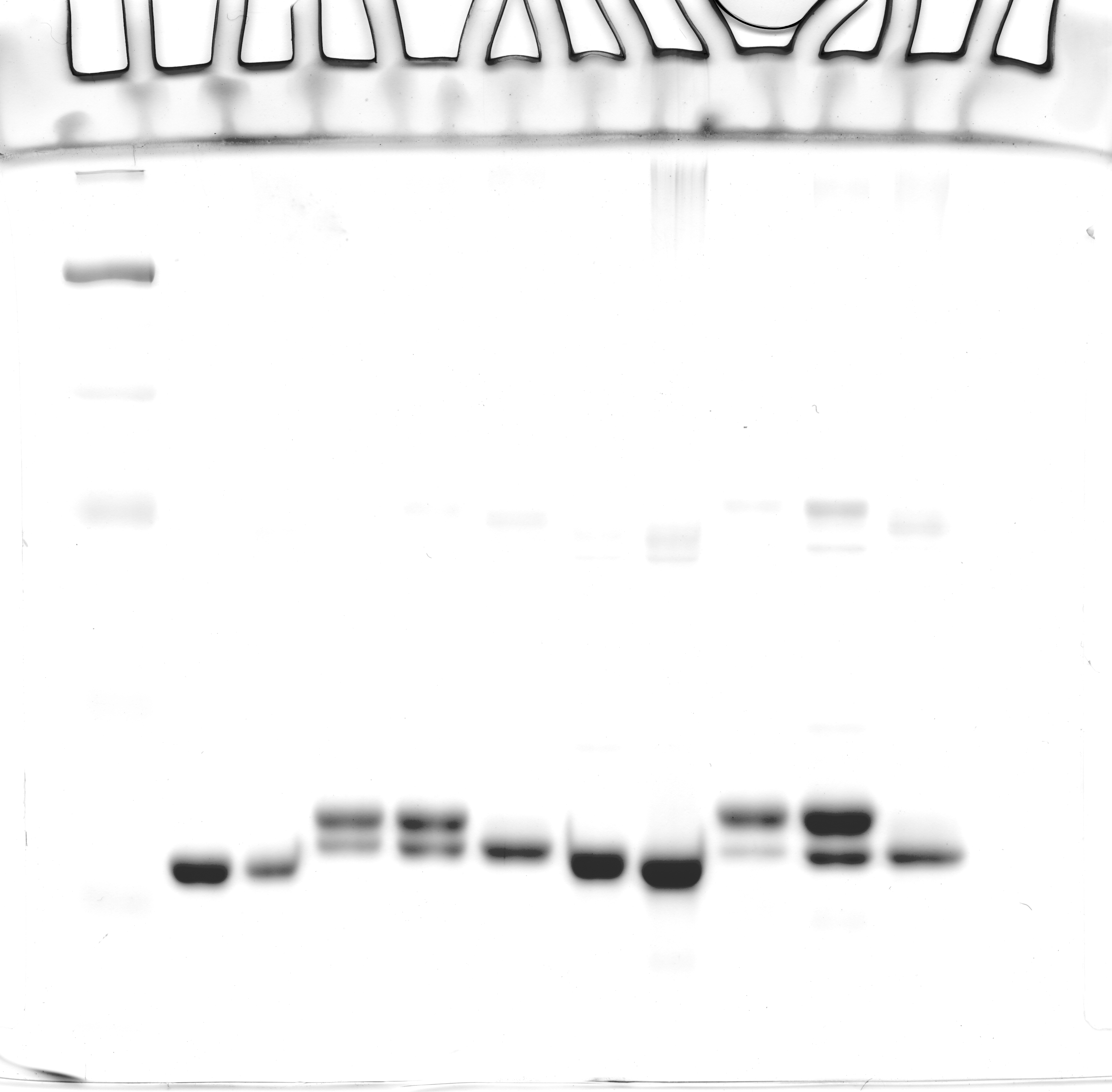

Supplement: Figure 1—source data 2. [file elife-106228-fig1-data2.zip › Fig1D-source2-rear.jpg]

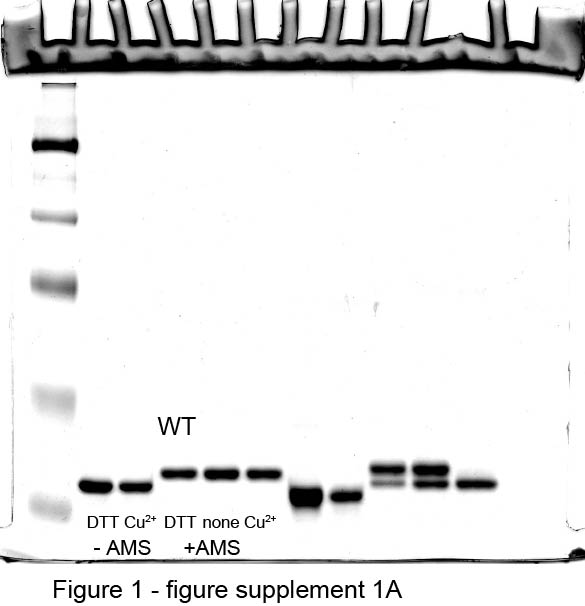

Supplement: Figure 1—figure supplement 1—source data 1. [file elife-106228-fig1-figsupp1-data1.zip › Fig1-suppl1-source1-WT-label.jpg]

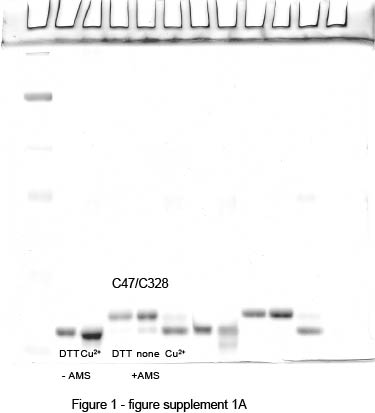

Supplement: Figure 1—figure supplement 1—source data 1. [file elife-106228-fig1-figsupp1-data1.zip › Fig1-suppl1-source1-front-label.jpg]

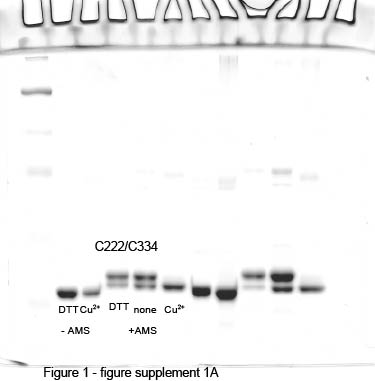

Supplement: Figure 1—figure supplement 1—source data 1. [file elife-106228-fig1-figsupp1-data1.zip › Fig1-suppl1-source1-rear-label.jpg]
